# Can smoking cessation be taught online? A prospective study comparing e-learning and role-playing in medical education

**DOI:** 10.5116/ijme.5ff9.bccc

**Published:** 2021-01-28

**Authors:** Elias Lauerer, Elena Tiedemann, Thomas Polak, Anne Simmenroth

**Affiliations:** 1 Department of General Practice, University Medical Centre Würzburg, Germany; 2 Department of Psychiatry, Psychosomatics and Psychotherapy, Center of Mental Health, University Medical Centre Würzburg, Germany

**Keywords:** Medical education, e-learning, smoking cessation, objective structured clinical examination

## Abstract

**Objectives:**

We compared the effect of different didactic formats - e - learning and role-playing - on medical students' knowledge and counselling skills in smoking cessation training.

**Methods:**

At a German medical school, 145 third-year students were randomly allocated to attend an online course with video examples or an attendance course with role-playing. Students were trained in smoking cessation counselling according to the 5A's (ask, advise, assess, assist, arrange) for approximately 90 minutes. Practical skills were measured in an objective structured clinical examination (OSCE) and represent the primary endpoint of this prospective comparative study. Additionally, changes in theoretic knowledge were assessed by pre - and post - interventional questionnaires and a final written exam.

**Results:**

In the OSCE, overall scores were higher in the attendance group (Mdn=70.8 % vs. 62.8 %; U=119; p=.087, n=36), but a statistical advantage was only found in one single counselling sequence (“Assist”: Mdn=66.7 % vs. 51.4 %; p = .049) and the rating of the standardised patients (M=4.7 vs. 4.2 out of 5 points, t_(27.836)_=2.0, p=.028). Students’ results (n=130) from self-assessment and written exams suggest that both approaches are equally well suited to increase theoretical knowledge. The online course was more time efficient (90 vs. 73 minutes).

**Conclusions:**

Seminar and web-based training seem equally well suited for transferring knowledge and skills on tobacco cessation counselling. Considering their particular strengths, these two teaching approaches could be combined.

## Introduction

"It's easy to quit smoking. I've done it hundreds of times." This joke, credited to Mark Twain, reflects a daily struggle for many tobacco smokers. Tobacco-smoking is the most important preventable health risk in developed countries.^1^ Most smokers know about the risks of their health behaviour and wish to stop or reduce tobacco consumption.^2^^, ^^3^ Nearly half of all smokers keep trying to quit at least once a year.^4^ These attempts are mostly unassisted and thereby unsuccessful, with quitting rates in a low percentile range.^5^

Even though effective counselling methods and medical interventions exist, only 4% of smoking patients in Germany receive evidence-based advice by their general practitioner.^6^ In addition to structural barriers, a lack of education in smoking cessation counselling is a key factor associated with the poor counselling rates. The problem has its origins in undergraduate education as medical students worldwide report poor knowledge and skills in treating patients with nicotine dependence.^7^^-^^12^ Medical students should undergo skills-orientated tobacco-educational training to assist patients in the process of smoking cessation. When such a course is implemented in medical education, three main questions arise: How extensive will the course be, what are the main learning objectives, and which didactic methods should be used? This study focusses on the last subject and directly compares two different didactic approaches: an online course with video examples, and an attendance course with role-playing.

Lecturing is the most widespread format to teach about tobacco in Germany and worldwide.^12^^,^^13^ Studies suggest that enhanced didactic methods such as role-playing or web-based modules are more effective for tobacco-education training.^14^^-^^17^ However, the most efficient didactic method of teaching medical students about tobacco cannot be determined because only a few studies exist.^18^ Additionally, the term "web-based module" is widely used in the literature but includes heterogeneous teaching methods that can hardly be compared.^19^

Several studies show that the knowledge of counselling smoking patients can be successfully transferred in a web-based module.^20^^-^^25^ Teaching online promotes self-directed learning and offers highly flexible learning opportunities for medical students.^26^ On the other hand, simulating conversations in small-group exercises has also been shown to be an effective teaching method.^15^^, ^^17^ While students prefer interacting with standardised patients (SP), role-playing with fellow students is equally well suited to improve practical counselling skills.^27^^-^^30^ In conclusion, the didactic methods "online-course" and "role-playing" have each been proven to be successful, but to our knowledge, these two approaches have never been compared directly to each other in the field of tobacco education.

This prospective comparative study explores to which extent different didactic formats – online-course and role-playing – influence medical students' knowledge and counselling skills in smoking cessation training.

The primary endpoint of the study was a comparison of students' practical counselling skills, measured in an objective structured clinical examination (OSCE). We assumed that the counselling skills for students in the seminar group are better because they actively performed a role-play, while students in the online course group only passively watched a video. On that basis, our primary hypothesis "the seminar group achieves better counselling skills, represented as a higher overall OSCE score than the web-based group (H1)" was formulated.

Growth in theoretic knowledge and development of a sympathetic attitude towards smoking were secondary endpoints. These items were assessed by pre - and post - interventional questionnaires and a final written exam. Systematic reviews suggest that theoretical knowledge can be conveyed comparably effectively through e-learning and traditional teaching methods.^31^^,^^32^ Therefore, the secondary hypothesis "regardless of the form of teaching, an improvement in knowledge about smoking is achieved (H2)" was formulated. As we assumed the same effect for students' attitude the other secondary hypothesis was that "regardless of the form of teaching, participants are sensitised to the problem of tobacco addiction and develop an appropriate attitude (H3)".

## Methods

### Study design and participants

This prospective comparative study was performed at the University of Würzburg, Germany, during the winter semester of 2018/2019. The whole cohort of third-year medical students (n=145) was enrolled and randomly sorted into two groups. While learning objectives and teaching content were widely congruent in both groups, different didactic methods were used for knowledge transfer: Students in group 1 attended a seminar with role-playing. Students in group 2 completed an online course with a video example.

The newly designed smoking-cessation training was implemented in the existing teaching module on "prevention and health promotion". This is a compulsory subject in medical students' education at the University of Würzburg. Students' knowledge of the course content was assessed in an obligatory written exam at the end of the sixth semester. When considering ethical issues, we became aware that one didactic format might be more successful in transferring knowledge, which could lead to an advantage for this group in the written exam. These considerations and the study plan were presented to but waived from the submission by the ethics committee of the faculty of medicine. The students council and the centre of general medicine at the University of Würzburg advocated the project.

### Measuring students' competencies

Objective and subjective criteria of knowledge, skills, and attitude were measured with different instruments of data collection: Questionnaires, questions in a written exam, and an objective structured clinical examination.

#### Questionnaires

Self-assessed data, collected in the questionnaires, were recorded and evaluated completely anonymously. In the first section, information on sociodemographic data (sex, age, and smoking status) was collected.

The following sections of the questionnaire surveyed students' attitude towards smoking (six items), their self-assessment of knowledge to treat tobacco addiction (four items and one multiple-choice question), and their self-assessment of counselling skills among smoking patients (three items). For each item, a statement was made (e.g., "I assume that tobacco smoking is an addiction disease") and students were asked to give their assessment on this statement using a five-level Likert scale: (1) strongly disagree, (2) disagree, (3) neutral, (4) agree, and (5) strongly agree. In the multiple-choice question, four widely used therapeutic options were presented (behavioural therapy, nicotine replacement therapy, drug therapy with Varenicline, and drug therapy with Bupropion). Students were asked to state which of the presented therapy options were familiar to them.

#### Written test

Objective knowledge was assessed by three multiple-choice questions that covered epidemiology, nicotine dependence, and the 5A model in the written exam on "prevention and health promotion" at the end of the semester.

#### OSCE

Practical counselling skills were assessed in a one-station OSCE. SPs of both genders were trained by one of the authors (AS) to perform the role of a young teacher who regularly smokes about 15 cigarettes a day. Because the teacher talks loudly in front of the class all day, the teacher suffers from voice problems. An ENT physician has diagnosed chronic laryngitis and recommended reducing the exposure to noxae such as alcohol or tobacco smoking. The teacher is motivated to quit smoking but worried and uncertain as several quitting attempts in the past were unsuccessful. For this reason, the teacher now consults the family doctor.

Students took up the role of the consulted family doctor. They had the task of conducting a short motivational consultation on smoking cessation, following the 5A approach. The time limit was set to 6 minutes. They had no demonstration material. The consultation room was equipped with two chairs, a table, and a clock.

Students' counselling performance was rated by two independent assessors using a standardised previously defined evaluation form. In a joint meeting some days before the OSCE, all raters were equally trained by the authors (AS, EL, and ET). Every pair of raters consisted of a graduate (primary care physician, psychologist) and an undergraduate (fourth-year medical student) assessor of different genders.

The rating scheme is based on existing OSCE forms but was adjusted to the specific counselling situation.^14^^,^^15^^,^^20^ It consists of 4 items on general communication, 1 item on recognising a link between complaints and smoking behaviour, and 14 items on cessation counselling according to the 5A's model (Table 1). For each item, a statement was presented (e.g., "Provides individually tailored benefits when smoking is stopped"). Considering the complexity and time aspects, items were rated on a dichotomous (yes or no; max. 1 point) or a three-level scale (completely fulfilled, partly fulfilled, and missed; max. 3 points). Additionally, raters and SPs were asked to assess their subjective impression of the counselling talk on a five-level Likert scale.

### Intervention: Content and course design

The web-based module and the seminar both focused on transferring application knowledge and practical counselling skills, following the 5A's approach. National and international guidelines recommend using the 5A's (ask, advise, assess, assist, and arrange) for brief verbal interventions in clinical practice.^33^^,^^34^ This counselling model is a valid and widely used guide that has been shown to increase rates of quit attempts and smoking cessations.^35^

Both interventions were designed as a one-time 90-minute course and comprised modules on knowledge and skills. Structure and content were identical. The same graphics and explanations were used in both interventions. Because of the different teaching methods, students in group 1 actively performed in a role-play, and students in group 2 passively watched a video example of counselling a smoking patient. The setting, patient's motivational status, and doctor's task in counselling were identical in the two didactic approaches.

Knowledge module (groups). After conveying basic knowledge on tobacco smoking (e.g., epidemiology, health consequences, physical and psychological aspects of tobacco addiction) and emphasising the role of doctors in tobacco cessation, different options of drug- and non-drug therapy were presented. The Fagerström test was introduced as an instrument to estimate patients' nicotine dependence.^1^ We focused on 5A's model, a motivational approach for short verbal interventions. Individual steps of counselling were explained, and phrases for patient-focused communication were suggested.^33^

Skills module - seminar (group 1): The classroom course was divided into small role-play groups of three students each. Each student was randomly assigned one of the prescribed roles of "doctor", "patient", or "observer".  Following the 5A's model, the "doctor" conducted a short counselling interview with the "patient", who is willing to quit smoking but needs professional assistance to successfully implement their vague plan. After seven minutes of counselling, the "observer" gave structured feedback (2 minutes). Three instructors were available for questions and provided additional feedback if necessary. The roles were changed for the second and third round of role-playing.

Skills module–online-course (group2): Participants watched a 14-minute video of a patient-physician conversation following the 5A's model.^36^ Single steps of this model were presented and directly linked to a realistic and concrete example for counselling a tobacco-smoking patient. The video could be halted or repeated individually. Comparable to the function of the "observer" in the role-play setting, a background voice in the video commented on the doctor's approach in the conversation.

Presentation tools: Graphics and central messages during the oral presentation in the seminar were shown in a standard PowerPoint presentation. In the online course, we used the presentation software Prezi for transferring knowledge and skills. As Prezi allows to produce mind maps, the overall structure of the course remains clear and comprehensible even if some topics are explained in detail. We proposed watching the slides in a linear sequence, but students were able to leave the suggested path if they preferred thinking and learning in a different individual way.

After the intervention, all students were given access to a pocket card. This pocket card summarises the 5A's model and the Fagerström test for nicotine dependence.

**Table 1:** Items rated in the objective structured clinical examination (OSCE)

Items: Student…		Achievable points	
General communication			9
	opens the counselling adequately	3	
	communicates the time frame of the conversation	1	
	speaks understandably and avoids medical language	3	
	conveys empathy	2	
Link between patient's symptoms and their tobacco consumption			2
Smoking cessation counselling according to 5 A's model			27
	Section Ask:		6
	- assesses smoking status	1	
	- assesses number of cigarettes per day	1	
	- calculates number of pack years	1	
	- asks about smoking behaviour after getting up	1	
	- acknowledges previous smoking attempts	2	
	Section Advise:		4
	- clearly advises patient to stop smoking	2	
	- provides individually tailored benefits of a smoke stop	2	
	Section Assess: determines patient's motivational status		4
	- by asking "are you ready to quit smoking?"	2	
	- by asking "do you think you can quit smoking?"	2	
	Section Assist:		9
	- advises to decouple smoking from personal rituals	2	
	- addresses the option of drug therapy	3	
	- advises to set a date	1	
	- advises to tell family and friends	1	
	- addresses personal triggers and challenges	1	
	- advises to remove all tobacco products	1	
	Section Arrange:		2
	- arranges an appointment after the smoking stop	1	
	- points out to further stop-smoking-services	1	
	Total points achievable		36
	Subjective mean global rating		Likert scale (1-5)
	SP rating		
	- ran counselling talk respectfully and professionally		Likert scale (1-5)
	- advised and motivated me very well		Likert scale (1-5)

### Data collection

During the introductory lecture for the winter semester 2018/2019, students were informed about the newly implemented teaching module and the accompanying research. They were asked for consent to participate in this educational study.

Every student in the seminar was given a certain date for the course (weeks 9, 10, and 11), while students in the online course group had access to the course for three weeks (weeks 9-11). Because resources were limited, the number of OSCE participants was calculated based on results from Stolz and colleagues.^15^ To provide a significance level of .05 and a power of 80%, the sample size for the OSCE was set at 19 students per group. Paper- and web-based questionnaires as well as the OSCE evaluation form were designed and digitalised using EvaSys 7.1.

Pre-interventional questionnaires were filled at the beginning of the semester (T1). Post-interventional questionnaires were filled directly after completing the intervention (T2). Students in the seminar were given printed questionnaires, students in the online-course followed a link to an online questionnaire covering the same content. OSCE (T3) and written exam (T4) were performed one week after all students participated in the intervention. To match the questionnaires at different points of time, students indicated a personalised six-digit code on each questionnaire. Students were only included in the analysis when a linkable pair of questionnaires at T1 and T2 was available, and OSCE results could be matched to an existing pair of questionnaires.

### Data analysis

The program SPSS 26.0 was used to conduct statistical analyses. Descriptive methods such as mean, median, and standard deviation were used to show data. Potential group differences in sociodemographic items for categorical data were assessed with the chi-square test (χ²), and continuous data such as age with Welch's t-test or one-way Welch's ANOVA, as appropriate.^37^

OSCE: Each item at the OSCE was evaluated by two examiners (graduate and undergraduate). The mean rating for each item for every OSCE participant was calculated. The means of each item were summed section by section (e.g., general communication, ask, and advise). When a value in one checklist, was missing only the second examiner's evaluation was considered. Sectional and overall percentage scores were calculated. To test the primary hypothesis, group differences in students' OSCE performance were addressed with the Mann-Whitney U-test. This test was chosen as data in the online group were not normally distributed (as determined by visual inspection) but homoscedastic. The same test procedure was used in previous studies on tobacco cessation training.^14^^,^^15^ Sociodemographic and OSCE score associations were addressed with Spearman's rho correlation. The inter-rater reliability between graduate and undergraduate raters regarding the overall OSCE score was measured using the intraclass correlation coefficient (ICC). ICC (1,1) was chosen because each subject was assessed by a different set of randomly selected raters.^38^

Questionnaire: To test our secondary hypothesis, pre-and post-ratings of self-assessed knowledge and attitude for every item were analysed using a paired t-test. Although some knowledge items violated the assumption of normality, this test was chosen as it is robust to nonnormality in large sample sizes (number of participants: 130).^39^

Learning-gain differences in knowledge and attitude between the seminar and web-based group were analysed with Welch's t-test.^37^ Learning gain was calculated based on a formula in which ceiling effects in knowledge growth are considered.^40^

Except for the primary directional hypothesis, all tests were two-tailed. Test results were assumed to be significant if p<.05. Effect sizes were calculated with Cohen's d.^41^^,^^42^

## Results

Out of a total of 145 eligible participants, one hundred and thirty-seven students (94.5%) submitted a questionnaire at T1, and 142 students (97.9%) submitted a questionnaire at T2. A match of completely filled out questionnaires at T1 and T2 was available for 130 students (89.7%). Response rates for questionnaires were similarly high in both groups: For the seminar, 94.4% (n=68) at T1 and 100% at T2, and for the web-based training, 94.5% (n=69) at T1 and 95.9% (n=70) at T2. Thirty-eight students performed in the voluntary OSCE (T3). One participant of each group was excluded from the analysis as the questionnaires could not be matched. The written exam was passed by 140 students (T4).

Sociodemographic characteristics and smoking status were distributed equally in both groups. The average age of the course participants was 23 years (SD=3). The students were 20 to 41 years old. The majority was female (70%; 90/129). Nine students (7%) reported that they currently smoked. There was a significant difference in age depending on smoking status, F_(__2, 12.738)_ = 6.225, p=.013. Post hoc analysis revealed that ex-smokers (n = 11) were significantly older (28 ±5years) than non-smokers (23±3, n=107) but not than smokers (25±4 years, n=9).

OSCE participants: Students attending the OSCE were significantly younger than their fellow students (M=23±2 vs. 24 ± 4 years, t_(__103.472)_=-2.129, p=.036). Seventy-five percent of the OSCE participants were female. While all participants of the seminar group were non-smokers, the web-based group included four participants with smoking experience (two smokers, two ex-smokers), χ²_(__1)_= 4.265, p=.104. OSCE participants in the online course group spent more time with the web-based training module than their fellow students (79 vs 72 min; t_(__25.021) _=1.036, p=.310). Overall results in the written test at T4 were comparable (t_(__62.414)_ =1.167, p=.248). Approximately 75% of the OSCE attendees prepared themselves in addition to the intervention, mostly for 20 minutes or less. Students in both groups prepared equally long (t_(__22.169)_=-0.910, p=.373).

### Primary endpoint: Communication skills

**Figure 1.**
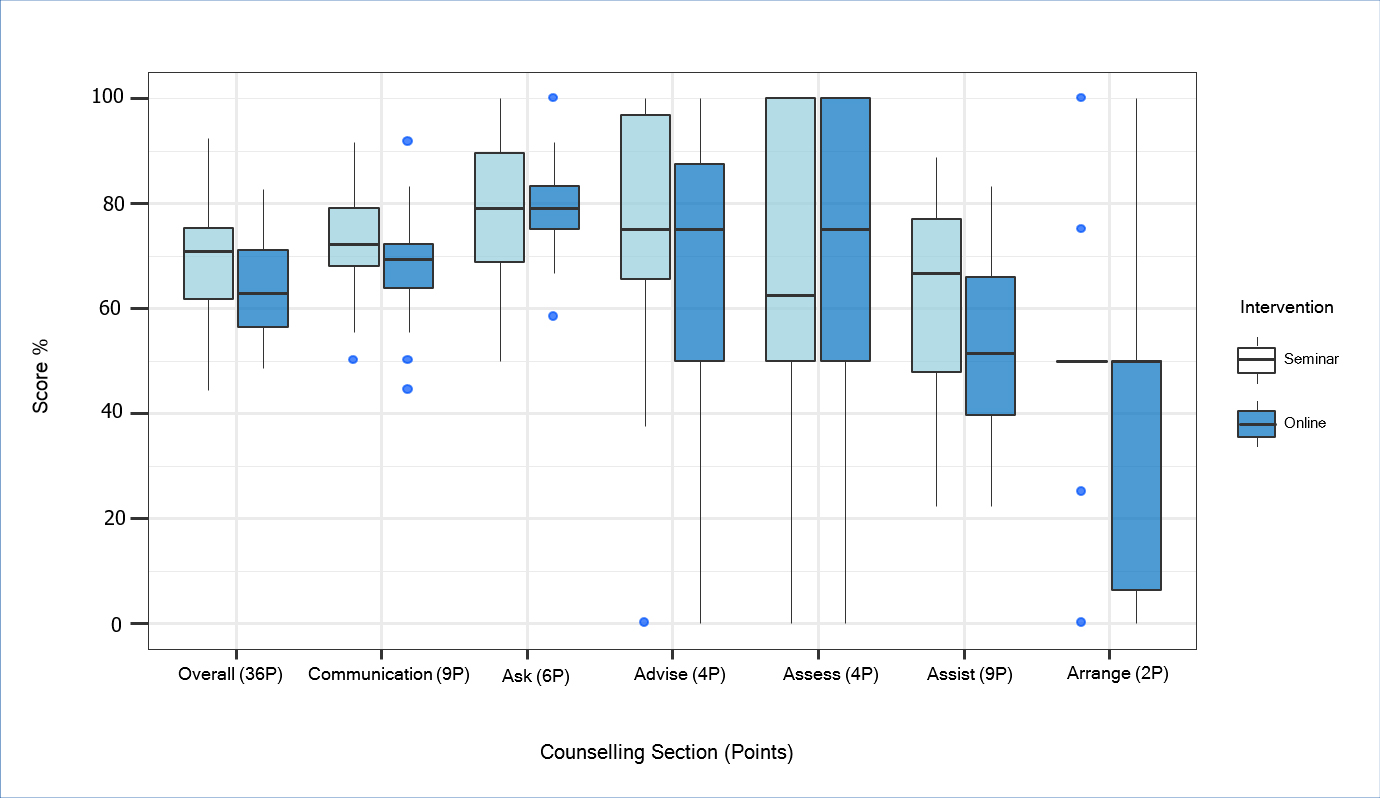
The distribution of the OSCE scores is displayed section by section as boxplots showing the median (horizontal line) and the interquartile range (IQR; lengths of the box). The vertical lines (whiskers) extend from the hinge to the largest/smallest value no further than 1.5*IQR from the hinge, values beyond these upper and lower bounds are considered outliers. Significant difference only in the section “Assist” (p = .049, n=36).

The overall OSCE scores were higher in the seminar group (Mdn = 70.8 %; IQR = 60.9%–76.2 %) than in the web-based group (Mdn = 62.8%; IQR: 55.9%–71.7%), but this difference was not significant (U=119, p=.087). When we analysed the individual sections of the 5A counselling model, the seminar group performed slightly better in four of the 5 As (exception: “Assess”). As shown in Figure 1, a significant difference was only found in the section “Assist” (Mdn = 66.7% (IQR: 47.2% –77.8%) vs. Mdn = 51.4% (IQR: 38.2% – 68.8%); U=110, p=.049, d= 0.57).

Overall school grades given by the raters were not different between seminar group (Mdn=2) and web-based group (Mdn=2), U=145, p=.582. There was a statistically significant strong positive correlation between the mean subjective global rating and the mean overall OSCE scores r_(__36)_=-.707, p<.001. Graduate and undergraduate raters assessed the students' OSCE performance equally (r_(__22)_=.704, p=<.001). The reliability measured by the ICC was 0.844 (95% CI 0.652 - 0.935), which indicated a good agreement.

The SPs rated counselling and motivational skills significantly better in the attendance-course group (M=4.7) than in the web-based group (M=4.2), t_(__27.836)_=2.0, p=.028, d= 0.67.

Students’ age (r_(__36) _=.183, p=.285) and sex (r_(36)_=.117, p = .495) was not significantly correlated with the OSCE scores. Because only the web-based group included participants with smoking experience, OSCE scores within this group were analysed depending on smoking experience: Overall OSCE scores for students without smoking experience (Mdn= 62.8%, n=14) and with smoking experience (Mdn=66%, n= 4) were not statistically significantly different, U=21, p= .457.

Students in the seminar group felt more confident in addressing tobacco smoking among patients, taking patients' smoking history and counselling them following the 5A's approach. Nevertheless, this self-assessed counselling skill did not vary significantly between the two groups (t_(__123.946) _= 1.321, p=.189).

### Secondary endpoints: Improvement of Knowledge and Attitude

Knowledge: Both interventions increased self-assessed knowledge in all five items significantly (p<.005, see Table 2). The didactic method had hardly any influence on knowledge growth: learning rates were equally high in both groups (see Table 3). Students reported significant learning gain in knowledge about risks, nicotine dependence, the 5A's model (two items) and the variety of therapy options (one item; multiple choice). 78.5% (n=102) of the students stated that they had learnt theoretic and application knowledge for counselling smokers mainly in this course. Students reported the highest learning success in the item "order of counselling steps (5A's model)". The lowest knowledge gain was achieved in the item on "risk awareness". However, more than one-third of the participants (37.7%, n=49) already fully agreed before they participated in the course about the risks of smoking. In the written test (T4), the majority answered two out of three questions correctly (42.1%, n=59). Only 17.1% (n = 24) of all students were able to answer all three questions correctly. Results were similar in both groups, t_(__137.917)_=-0.688, p=.493.

Attitude: The overall attitude towards smoking (six items) was not affected by course participation, t_(__126)_=0.072, p= .943. Even before they participated in the intervention, 120 students (93%) considered smoking to be a chronic addiction disease and recognised the important role of doctors in smoking cessation treatment. Nevertheless, after the course, only half of the students (n=72) were convinced that doctors could effectively influence patients' smoking behaviour. Consistency in attitude towards smoking was found in both groups. The didactic form had no influence, t_(__122.180)_=- 0.321, p=.749.

### Course evaluation

In addition to testing the primary and secondary hypotheses, the newly implemented course was evaluated. Both interventions were rated equally as “good”, (t_(__126.549) _=- 0.305, p=.761). Prior to the intervention, students in both groups preferred the online module to the seminar. After the intervention, more than 80% in both groups were satisfied with the didactic form they were assigned. Few students reported technical problems with the online module, all of which could be solved via e-mail support. Students in group 1 spent 90 minutes of educational time on the seminar, while those in group 2 completed the web-based training in 74± 25 minutes, t_(__53.000) _=4.795, p.001. Afternoon (39%) and evening (30%) were the preferred times for working on the online course.

**Table 2:** Self-assessed learning gain rating before and after the course

Learning gain	Pre		Post		t	p-value	d
	M	SD	M	SD			
Knowledge (5 items)							
Risk awareness	4.2	0.8	4.4	0.7	-3.0	.004	0.3
Nicotine dependence mechanism	2.5	1.1	4.2	0.8	-14.9	<.001	1.3
Familiarity with the 5A’s	1.2	0.6	4.3	1.3	-23.4	<.001	2.1
Order of counselling steps (5A’s)	1.1	0.3	4.4	0.7	-44.9	<.001	3.9
Number of familiar therapeutic options ^a^	2.0	0.6	3.8	0.6	-24.8	<.001	2.2
Counselling skills (3 items)							
Addressing tobacco smoking	3.0	1.1	3.9	0.9	-7.5	<.001	0.7
Taking patient’s smoking history	2.8	1.2	4.2	0.7	-11.6	<.001	1.0
Counselling talk according to the 5A’s	2.6	1.0	4.0	0.7	-14.1	<.001	1.2

Note: Scale ranged from 1 (completely disagree) to 5 (completely agree) significant if p < .05 significant results are in bold. df=129. a Multiple-choice question, 4 therapeutic options presented.

## Discussion

This prospective comparative study examines whether different teaching formats influence medical students' skills, attitude, and knowledge of smoking cessation counselling. By combining different instruments of data collection, we were able to measure students' learning success in the three competency levels "knows", "knows how", and "shows" according to Miller's pyramid.^43^ Self-assessment of knowledge and skills can be used as a valid parameter for pre-post comparison. It appears to be closely related to general self-attribution and is a relatively stable individual characteristic.^44^ Under this assumption, some people regularly tend to overestimate their performance, but as the discrepancy remains consistent over time, this becomes negligible in pre-post-comparison.^45^^,^^46^

The mean total OSCE score was not significantly higher in the attendance course group. Our primary hypothesis (H1) is, therefore rejected. From the statistical point of view alone, both teaching methods are equally well suited to transfer counselling skills.

**Table 3:** Comparison of self-assessed learning gain between groups

Category	Learning Gain (%)		t	df	p-value
	Group 1 (Seminar)	Group 2 (Online)			
Knowledge	36.6	34.3	0.881	93.312	.381
Counselling skills	24.9	20.2	1.321	123.946	.189
Attitude	-1.3	-0.6	- 0.321	122.180	.749

Note: Data are presented as mean percentage of the learning gain per category; p-values analyse group differences and refer to the Welch's t-test

On the other hand, the overall appraisal of the results suggests a possible advantage for the seminar with role-playing. Students who attended the seminar (group 1) performed better in the comprehensive counselling section "Assist". It appears to be particularly important for successful counselling that this section is covered. A case-control study with more than 3000 patients concluded that covering the sections "Assist" and "Arrange" increases the rates of successful smoking cessations in particular.^47^ The SPs rating of the students' counselling performance was significantly higher in group 1. The SPs rating is an important parameter to improve the quality and reliability of the OSCE assessment process.^48^ Finally, students who attended the seminar appeared to have gained slightly more confidence in counselling smokers. In summary, internal (self-assessed skills) and external (OSCE performance) assessment of practical skills was widely congruent and suggests a higher learning success for the seminar group. Even though these differences in practical counselling skills are only additions to the primary hypothesis, they represent a meaningful advantage in students' counselling skills that we attribute to the didactic concept "seminar with role-playing".

The use of a second independent OSCE rater provided no additional information. Undergraduate medical students can act as OSCE raters when they have been trained adequately, and the rating scheme is clear. These findings are consistent with the results in the research literature.^49^

Results reveal that the 90-minute intervention, regardless of the didactic form, leads to significant growth in knowledge. The online course was more time-efficient. Students' attitude towards smoking and their comprehension of the doctors' role in smoking cessation was favourable before the intervention and remained consistent. Our undirected secondary hypothesis (H2) is, therefore accepted. This result stands in accordance with findings from other studies on tobacco education training and a meta-analysis comparing Internet and no-internet interventions.^15^^,^^20^^,^^32^ Students are open towards the new teaching approach. This is shown by their preference for the web-based module, which they used effectively to gain knowledge and develop counselling skills. In accordance with results from other studies, Prezi can be used successfully as a well-working presentation tool in higher education.^50^^-^^52^

Following a skills-orientated approach, teaching time alone is not paramount in assessing the usefulness of didactic modules. While most other modules on tobacco education are more time-consuming, our 90-minute module can be easily implemented in existing medical curricula.^14^^,^^17^

### Strength and limitations

All third-year medical students were enrolled, and response rates were continuously high. The participating group, therefore, appears to be representative for the entire cohort of students this year. The two interventions were conducted simultaneously, and all participants were equally advanced in their studies. As the teaching module was newly implemented, students' opinion and exam preparation were not influenced by more advanced fellow students. Except for the post-interventional questionnaire (T2), all data in both groups were collected at the same time. Information-sharing between students therefore hardly affects the results.

The teaching times in both interventions in our study are comparable. We collected exact data on time spent during the online course. Some studies avoid making an exact statement on the time spent online because no data have been collected or because educational times vary widely between different didactic approaches.^15^ It often remains unclear whether possible differences in knowledge and skills are due to different teaching methods or to additional educational time.

Our study has some limitations. As the curriculum at the Würzburg medical school only allows offering events to a clearly defined semester, the maximum sample size was predetermined by the 145 students in the current semester. Out study was conducted with a relatively homogenous group of students at a single medical school. The enrolled group is characterised by a high portion of female and non-smoking students. A comparison to other sociocultural environments or healthcare systems might therefore be limited.

Students in the online course watched a video in English of a counselling talk, but the role-play was conducted in German language. In the OSCE all students were required to express themselves in German. Students in the online-course group had heard some key phrases only in English before. This might have influenced their OSCE performance negatively.

The voluntary OSCE was performed with 19 students per group, a relatively small sample size. Students participating in the OSCE were significantly younger. A selection bias of highly motivated students into the voluntary OSCE group cannot be ruled out. The sample size calculation was based on a study of Stolz and colleagues^15^ that used a similar design but had one crucial difference: Stolz and colleagues compared a group (n = 35) using a web-based module alone to a group (n = 31) using a web-based module plus additional role-playing for tobacco cessation training. This second group spent considerably more learning time, which might have contributed to a great difference between the groups and to the large effect size.

In our study participants completed the same tobacco cessation training, either as a web-based module or as a seminar. This might have reduced the differences between the two groups' OSCE performance compared to the study of Stolz and colleagues. It can thus be suggested that our sample size was too small, and our study was underpowered. Based on a calculated effect size of d=.44 and a sample size of 18 participants per group, the achieved power was indeed only 36%. To gain a power of 80%, all 130 students should have been included.

Finally, we did not address the problem with multiple comparisons. If the level of significance were adjusted to the number of p-values calculated, some group differences, for example of the item "Assist", would no longer be assumed significant.

### The implication for future research

Gaining knowledge and practical counselling skills is essential for treating patients who smoke and should become an integral part of undergraduate medical education. Because curricula are crowded, and the opportunities of web-based learning grow constantly, medical schools need to determine the best-suited didactic approach to teach smoking cessation successfully and efficiently. Results of this prospective study show that teaching online is a successful and time-efficient didactic method in smoking cessation training.

The inverted classroom model might include specific advantages of both didactic formats. Following this approach, factual knowledge is transferred online and can be trained and applied in a subsequent attendance phase.^53^ Because our two teaching modules are well examined and are proven to be effective, they might be adjusted and combined into an inverted classroom course. The effectiveness of this teaching approach could be compared to results from our study.

Finally, the long-term effects of these two teaching interventions are still unknown. To this end, we are currently working on a follow-up study to assess long-term results of students' knowledge and skills in smoking cessation two years after completing the module.

## Conclusions

Our results suggest that both didactic methods, attendance course and web-based training, are equally well suited for transferring knowledge and skills on tobacco cessation. Each teaching method has particular strengths: The online course is characterised by time efficiency and students' preference while attending the seminar leads to a higher self-confidence in skills and a better SPs rating of counselling performance. Considering their particular strengths, these two teaching approaches could be combined.

### Acknowledgements

We would like to thank all students and simulation patients for their participation in this study, especially in the OSCE. We would also like to thank Christoph Müller, who helped create the questionnaires.  This publication was supported by the Open Access Publication Fund of the University of Wuerzburg. Other expenses were covered by the Department of General Practice, University Medical Centre Würzburg.

### Conflict of Interest

The authors declare that they have no conflict of interest.
